# *Citrus* Physiological and Molecular Response to Boron Stresses

**DOI:** 10.3390/plants11010040

**Published:** 2021-12-23

**Authors:** Lin-Tong Yang, Jun-Feng Pan, Neng-Jing Hu, Huan-Huan Chen, Huan-Xin Jiang, Yi-Bin Lu, Li-Song Chen

**Affiliations:** 1 College of Resources and Environment, Fujian Agriculture and Forestry University, Fuzhou 350002, China; talstoy@163.com (L.-T.Y.); 1200807017@fafu.edu.cn (J.-F.P.); 3200831018@fafu.edu.cn (N.-J.H.); huanhuanchen27@163.com (H.-H.C.); yibin.07@163.com (Y.-B.L.); 2 College of Life Sciences, Fujian Agriculture and Forestry University, Fuzhou 350002, China; jianghx@163.com

**Keywords:** *Citrus*, boron stress, B deficiency, B excess, tolerance

## Abstract

Since the essentiality of boron (B) to plant growth was reported nearly one century ago, the implication of B in physiological performance, productivity and quality of agricultural products, and the morphogenesis of apical meristem in plants has widely been studied. B stresses (B deficiency and toxicity), which lead to atrophy of canopy and deterioration of *Citrus* fruits, have long been discovered in citrus orchards. This paper reviews the research progress of B stresses on *Citrus* growth, photosynthesis, light use efficiency, nutrient absorption, organic acid metabolism, sugar metabolism and relocation, and antioxidant system. Moreover, the beneficial effects of B on plant stress tolerance and further research in this area were also discussed.

## 1. Introduction

Boron (B), an element essential for the growth, differentiation and reproduction of plant, exists in mineral such asborax, borosilicate, albite, brucite, colemanite, cristobalite, datolite and suanite, etc. B is widely distributed both in the Earth’s crust (from 5 mg kg^−1^ in basalts to 100 mg kg^−1^ in shales), in topsoil(from 4 mg kg^−1^to 145 mg kg^−1^) and in the ocean(~4.5 mg L^−1^) as borate [1,2]. The well-established function of B in plants is to form the covalent bridge between pectin molecules by its cross-linking with pectic domain rhamnogalacturonan II(RG-II) in cell wall by the formation of RG-II-B diolester [3,4,5]. Such bridging decreases cell wall porosity, modifies cell wall biomechanical properties, plasticity and thickness, and regulates pectin assembly and plant development [3,6,7,8,9,10]. Borate ion and boric acid are highly soluble, hence B deficiency has been reported in both nature and agricultural practice, especially in areas with high temperature and high rainfalls [1]. The conventional method to solve B deficiency in field is to apply B-containing fertilizers. However, the heavy and long-term application of B fertilizer, mainly borax, and B-rich irrigation water, sewage sludge, or fly ash, frequently lead to B excess in agricultural practice [11]. Therefore, B deficiency and B excess could be both found in agricultural production.

B deficiency generally impairs cell elongation rather than cell division in growing tissues of plants. Typical B deficiency symptoms include inhibition of root cell elongation and pollen tube elongation, development of leaf interveinal chlorosis, enlargement, cracking and lignification of main lateral veins [12,13]. Since the first report of the essentiality of B required for the normal grow than development of citrus by the use of sand cultures nearly one century ago [14], B deficiency is frequently observed in *Citrus* orchards, and is responsible for loss of productivity and poor fruit quality [15,16,17]. Under B-deficient conditions, the numbers of parenchyma cells in the vascular bundles of the leaf and fruit mesocarp increased significantly, whereas the lengths of xylem vessels in this tissue were both significantly lower than the corresponding values in control ones [18]. Anatomic analysis result showed that the cell wall was thicker and the inner vessel diameter was smaller in the B-deficient treatment than in the control ones. Such structural changes eventually reduced the function of the vessels in absorption and transportation of mineral nutrients, resulting in a limited nutrient supply and reduced growth of *Poncirus trifoliata* [19]. Sweet orange grafted on Swingle citrumelo sustained better growth by combining higher B absorption and root growth as well as better organization of xylem vessels under low B conditions. Under B toxicity, Swingle rootstock would improve water transport and favor plant growth by reducing anatomical and ultrastructural damage when compared with plants grafted on Sunki mandarin [20]. Despite the remarkable function of B on cell wall, B has been shown to function in the maintenance of plasma membrane stability and consequently regulate PM H^+^-ATPase activity generating a driving force for nutrient influx at the root level, and hence affecting nutrient uptake by *Citrus* roots [21]. B deficiency decreased the concentrations of chlorophylls (Chls) and carotenoids, CO_2_ assimilation, impaired photosynthetic electron transport chain, and significantly increased intercellular concentration and the concentrations of photosynthates such as glucose, fructose and starch in *Citrus* leaves [13,22]. Such altered carbohydrate metabolism inevitably affected other biological processes, such as amino acid metabolism, organic acid metabolism, and secondary metabolism. According to our previous study, B deficiency significantly upregulated the respiration, organic acid metabolism, amino acid and total phenolics biosynthesis in *Citrus sinensis* leaves, whereas downregulated these metabolisms in *C. sinensis* roots [23]. Similarly, B deficiency altered the key metabolites in glycolysis, amino acid metabolism and tricarboxylic acid (TCA) cycle, which was supposed to be associated with the inhibition of root elongation and the inflation of root tips in *P. trifoliata* under B starved condition [24]. Similar to B deficiency, B excess also decreased the CO_2_ assimilation and stomatal conductance, and increased intercellular CO_2_ concentration in *Citrus* leaves [25]. B toxicity led to alterations in pectin network crosslinking structure, destruction of cell wall integrity, chloroplast disintegration and plastoglobulus accumulation in *P. trifoliata* leaves, revealed by Fourier transform infrared spectroscopy (FTIR) and Transmission electronmicroscopy (TEM) analysis, respectively. B toxicity decreased the concentrations of proteins and cellulose, and increased carbohydrate concentration and starch grains in *P. trifoliata* leaves [26]. Both B deficiency and B excess could eventually lead to atrophy of canopy and deterioration of *Citrus* fruits [15]. 

In recent years, the molecular responses of plants to B deficiency or B excess were investigated to understand the adaptive mechanisms of plants to B stresses. For example, by using iTRAQ technique, a total of 164 up-regulated and 225 down-regulated proteins were identified in B-deficient *C. sinensis* roots [17]. Two-dimensional electrophoresis (2-DE) based MS approach reported that B excess increased the abundances of proteins involved in antioxidation, detoxification, proteolysis and cell transport in leaves and roots of *C. sinensis*, which might confer higher B-toxicity tolerance of *C. sinensis* than that of *C. grandis* [27,28]. Transcriptomic analysis showed that the expression levels of genes related to a broad biological processes from B uptake to plant development regulation and cell cycle, were altered by B stress in *Citrus* plants [17,29,30,31,32,33,34]. Furthermore, post-transcriptional regulation profile of gene expression by miRNAs was also investigated by Illumina sequencing in B-deficient *C. sinensis* leaves and roots [29,35].

Scion-rootstock combination studies demonstrated the different abilities of B uptake and root-to-shoot translocation existed among several key rootstocks, such as *P. trifoliata*, Swingle citrumelo, Carrizo citrange (CC), Cleopatra mandarin (CL), *C. macrophylla* (CM) and sour orange (SO), used in *Citrus* plantation [22,36,37,38]. Results obtained in these studies provided a scientific reference to select proper rootstock to cope with possible B deficiency or B excess conditions [20,21,39]. In addition, for ameliorating the adverse effects of B excess, some compounds, such as nitric oxide (NO), chitosan, methyl jasmonate (MeJA), and melatonin have been applied to plants [37]. Previous study showed that leaf Ca, Mg and B concentrations decreased with K rates in sweet orange, indicating that interactions of B with other nutrients, such as K, Ca and Mg, were important for the nutrient management of orchards on different rootstock varieties [40,41]. Although the underlying mechanism was not fully elucidated, the application prospect of those elements or compounds in *Citrus* plantation could be expected in the future. Future efforts could pay attention to some biomarkers or key proteins such as *Cs*NIP5;1, sorbitol-6-phosphate dehydrogenase, laccase 4 (LAC4), LAC7 and LAC17, which played vital roles in pectin biosynthesis, B transport and lignin biosynthesis, respectively, in *Citrus* plants cultivated in B problem locations [42,43,44,45].

B deficiency causes fruit drop and gum pockets in the peel. It sometimes occurs when farmers use only high-analytical grade fertilizers. Sinceit leaches readily, B should be applied every year either as a soil or foliar application [16]. Limited studies showed that soil application was more efficient than foliar application for the *Citrus* plants, which was important for best management strategies of B application. About 20 to 35% of B content in the new parts of orange tree came from plant reserves. B mobility within the plant was influenced by its nutritional status [46,47]. In contrast, management of B fertilizer and irrigation water, give a challenge to production practitioners in maintaining suitable B supply for *Citrus* trees. As a matter of fact, in China, B-toxicity occurs in some *Citrus* orchards. Up to 74.8% and 22.9% of pummelo (*C. grandis*) orchards in Pinghe, Zhangzhou, China, are excess in leaf B and soil water-soluble B, respectively [48]. This fact highlights the importance of understanding the role of B in *Citrus* tree growth and fruit production. In this paper, we reviewed the research progress of B stresses on *Citrus* growth, photosynthesis, light use efficiency, nutrient absorption, organic acid metabolism, sugar metabolism and relocation, and antioxidant system. Moreover, the beneficial effects of B in *Citru*s tress tolerance and further research in this area were also discussed.

## 2. Physiological Responses to B Stress in *Citrus*

**Figure 1:**
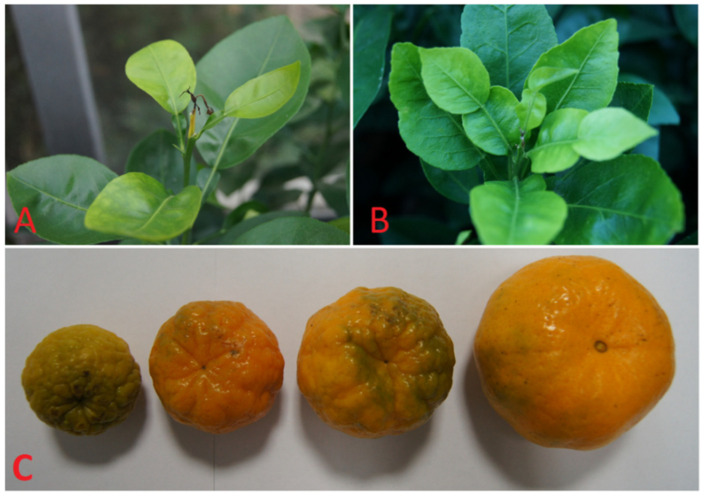
Symptoms of B deficiency in the leaves of *C. sinensis* (**A**) and *C. grandis* (**B**), and the fruits of “Ponkan” (*C. reticulata*) (**C**). Seedlings of *C. sinensis* (**A**) and *C. grandis* (**B**) were treated with 0 μM H_3_BO_3_ for 15 weeks, respectively. Fruits of “Ponkan” were collected from *Citrus* orchard in Yongchun county, Quanzhou, Fujian province, China (**C**).

Once absorbed by the roots, B is primarily transferred to mature leaves through xylem cells driven mainly by transpiration, except for some sugar alcohols (i.e., mannitol and sorbitol)-producing species such as *Pyrus*, *Malus*, *Prunus*, *Allium* and *Brassica*, many plants including *Citrus*, phloem mobility of B is limited [19,49]. Thus, the symptom of B deficiency was firstly developed in tender leaves and new shoot of *Citrus* plants, leading to dieback of terminal growth (Figure 1A,B). Along with the duration of B deficiency, the most apparent symptoms including spot-like chlorosis, coarsening, crimping, enlargement and cracking of leaf veins were observed in *C. sinensis* and *C. grandis* leaves [18,25].

It has been reported that B-deficiency leads to misshapen, small and hard fruit in *C. sinensis* [15,50]. There is limited information about the effects of B deficiency on *Citrus* fruits. According to available literatures and our observation, the symptom of B deficiency on *Citrus* fruits varied in different *Citrus* species, at least on the flavedo of fruits, for example, B deficient fruits developed asymmetrically in longitudinal sections, and brown secretions could be seen in the fruit peels of pummelo fruits [51]. During fruit expanding stage, B deficiency caused rough flavedo, small-size, chlorosis and abscission on “Ponkan” (*C. reticulata*) in *Citrus* orchard located in the southeast of Fujian province, China (Figure 1C).

**Figure 2:**
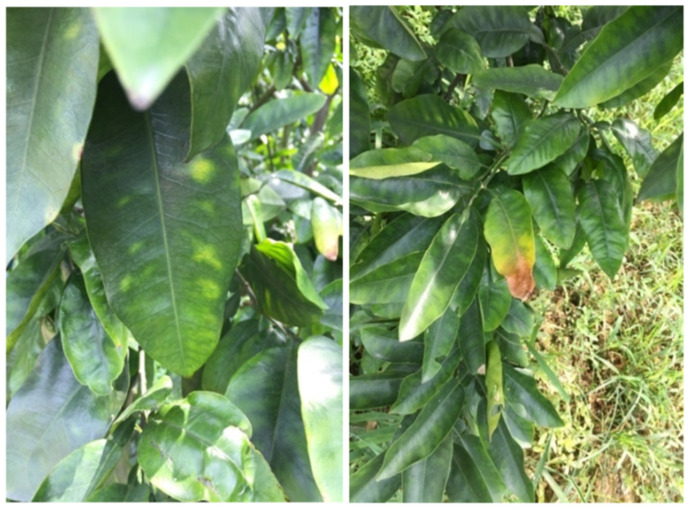
Leaf symptoms of B toxicity on pummelo (*C. grandis*) in field.

As B has limited mobility and can only be supplied to growing tissues in the xylem, B uptake followed the passive water flux from roots to leaves and accumulated especially where leaf veins terminate [49]. Under B excess condition, tip yellowing of the lower part leaves, followed by marginal and interveinal chlorosis were observed in mature leaves. Along with the duration of B excess, these chlorotic leaves became necrotic and fall off prematurely (Figure 2) [25].

**Figure 3:**
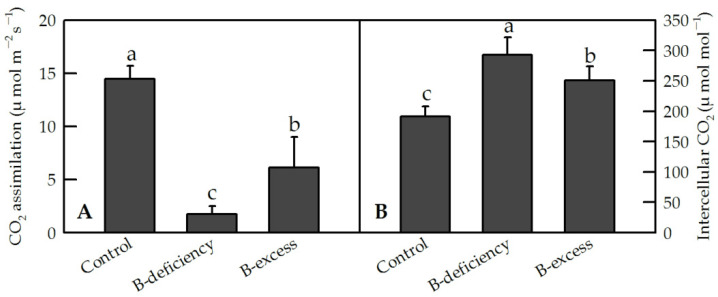
Effects of B deficiency and B excess on leaf CO_2_ assimilation (**A**) and intercellular CO_2_ concentration (**B**) in *C. sinensis*. Seedlings of *C. sinensis* were treated with 10 (Control), 0 (B-deficiency) and 400 μM (B-excess) H_3_BO_3_ for 15 weeks, respectively. Bars represent means ± SD (*n* = 4). Differences among the treatments were analyzed by ANOVA. Different letters above the bars indicate a significant difference at *p* < 0.05.

Both B deficiency and B excess could induce the degradation of Chls, leading to a compromised ability to process incident light by photosynthesis in plants, which were eventually more susceptible to oxidative damage [52,53]. B deficiency decreased Chl *a*, Chl *b* and carotenoid concentrations, but increased the ratio of Chl *a*/Chl *b* in *C. sinensis* leaves [13]. Chl *a* fluorescence (OJIP) transients from B-deficient *Citrus* leaves displayed a positive L- and K-bands, an increased initial fluorescence (F_o_) and a decreased maximum quantum yield of primary photochemistry (F_v_/F_m_), meaning that the PSII units are less grouped (stable) or less energy is being exchanged between independent PS II units, and that the oxygen-evolving complex (OEC) was damaged in these leaves. Moreover, B-deficiency-induced photoinhibition of PSII led to reduced use of light energy in electron transport and increased accumulation of excess light energy, which eventually elevated the production and accumulation of reactive oxygen species (ROS) [13,25]. In fact, B-deficiency-induced impairment of photosynthetic electron transport and reduction of CO_2_ assimilation might be caused by feedback inhibition, as B deficiency significantly increased intercellular CO_2_ concentration and the concentrations of photosynthates such as glucose, fructose and starch in *Citrus* leaves (Figure 3) [13,25]. Although, the primary mechanisms of B’s roles in photosynthesis are unknown, B could impact the biological functions of internal membranes in chloroplast by disturbing thylakoid electron transport and the energy conversion rate, resulting in photoinhibition [54]. Similarly, the repression of photosynthetic enzymes, such as ribulose-1,5-bisphosphate carboxylase/oxygenase (Rubisco), NADP-glyceraldehyde-3-phosphate dehydrogenase (NADP-GAPDH) and stromal fructose-1,6-bisphosphatase (FBPase), were also observed in B-deficient *Citrus* leaves [13]. B-deficiency significantly reduced the concentrations of Chls and carotenoids, and increased the concentrations of total soluble sugar and lignin in ‘Newhall’ navel orange. Moreover, B-deficiency substantially down-regulated the expression levels of photosynthesis-related genes, and induced the expression levels of genes related to Chl decomposition, glucose synthesis and lignin synthesis, but significantly inhibited the expression of carotenoid synthesis-related genes in ‘Newhall’ navel orange [22].

On the other hand, B toxicity decreased CO_2_ assimilation and stomatal conductance, but had no impact on intercellular CO_2_concentration. According to the stomatal limited theory in photosynthesis, B-toxicity-induced decrease in CO_2_ assimilation of *C. sinensis* and *C. Grandis* was mainly caused by non-stomatal factors. JIP test indicated that the damage of photosynthetic electron transport induced by B stress was more severe by B toxicity than by B deficiency in *C. grandis* leaves [25]. In addition, the typical B-toxic symptom only occurred in B-toxic *C. grandis* leaves, and no visible symptoms were observed in B-toxic *C. sinensis* leaves except for very few seedlings [27]. 

B-toxicity-induced decreases of seedlings growth, leaf CO_2_ assimilation, pigments and total soluble protein concentrations and increase of leaf malondialdehyde (MDA) concentration were less pronounced in *C. sinensis* than in *C. grandis* seedlings. These results draw the fact that *C. sinensis* is more tolerant to B-toxicity than *C. grandis* [25,27,31,55]. Sweet orange scions grafted on Swingle were more tolerant to both B deficiency and toxicity than those on Sunki, as revealed by higher shoot and root growth, and less reductions of both whole plant leaf-specific hydraulic conductance and leaf CO_2_ assimilation [20]. Moreover, Huang et al. [55] reported that leaves from B-toxic *C. grandis* seedling had higher free B and lower bound B as compared with those from *C. sinensis*. B toxicity led to irregular cell wall thickening in leaf cortex cells and phloem tissue of both *C. grandis* and *C. sinensis* leaves, but exocytosis only occurred in *C. sinensis*. In contrast, B toxicity induced programmed cell death (PCD) of phloem tissue was only observed in *C. grandis* leaves. They concluded that the higher B-toxicity tolerance of *C. sinensis* than *C. grandis* could be attributed to the lower leaf free-B concentration and higher bound B concentration in the former ones [55]. 

ROS, including singlet oxygen (^1^O_2_), superoxide anion (O_2_^·-^), hydrogen peroxide (H_2_O_2_), and hydroxyl radical (HO^·^), are partially reduced or excited forms of atmospheric oxygen, which can be produced in nearly every sub-cellular compartment. They are the unavoidable toxic byproducts of aerobic metabolism, and function as signaling molecules as well [56,57]. Increments of ROS production have been found in *Citrus* plants encountering various environment stresses including phosphorus deficiency, aluminum toxicity, magnesium deficiency, manganese toxicity and B stresses [28,58,59,60,61,62]. As a strategic response, a large group of antioxidant molecules, such as amino acids, carotenoids, flavonoids, ascorbate (ASC) and reduced glutathione (GSH), and a series of antioxidant enzymes, for example, superoxide dismutase (SOD; EC 1.15.1.1), catalase (CAT; EC 1.11.1.6), ASC peroxidase (APX; EC 1.11.1.11), monodehydroascorbate reductase (MDAR; EC 1.6.5.4), dehydroascorbate reductase (DHAR; EC 1.8.5.1) and glutathione reductase (GR; EC 1.6.4.2), were found in plants [57]. Characterized by reacting rapidly with H_2_O_2_ and their oxidized forms being regenerated by high-capacity reductases and associated systems, ASC and GSH are key players in antioxidant system and measured as represent of antioxidant compounds in many circumstances [63]. Both ASC and GSH concentrations were increased by B-deficiency but were decreased by B-excess in *C. sinensis* leaves. B-deficient leaves had higher SOD, APX, MDAR, DHAR and GR activities, lower CAT activity. B excess leaves had higher SOD, APX, and GR activities, lower MDAR, DHAR and CAT activities. B deficiency significantly increased O_2_^·-^ generation, MDA concentration and electrolyte leakage in *C. sinensis* leaves. B-deficient leaves displayed higher or similar activities of antioxidant enzymes compared to B-excess ones. Although both B-deficient and -excess leaves remain high in activity of antioxidant enzymes, the up-regulated antioxidant system could not provide sufficient protection to these leaves against oxidative damage, as MDA concentration was simultaneously increased by B deficiency and B excess [13,25]. Similarly, B deficiency elevated O_2_^·-^ and H_2_O_2_ generation and electrolyte leakage in tea plants. The activities of APX, CAT, peroxidase (POD; EC 1.11.1.7) and SOD were increased in B deficient plants. Simultaneously, transcripts of the antioxidant enzymes were up regulated under B deficiency. By and large, the authors concluded that B deficiency intensifies ROS generation, but the antioxidant system could not provide for an adequate protection from oxidative damage, as the concentration of MDA in tea leaves was increased by B deficiency [64]. Simón-Grao et al. [65] investigated the responses of different *Citrus* rootstock to B excess. SO rootstock proved to be more tolerant to B toxicity than CC rootstock. Such result was attributed to less accumulation of B in SO leaves, as its roots might sense the external B concentration and restrict the uptake and relocation of B into upwards the shoots. In addition, SO was suggested to encounter B-toxicity-induced oxidative stress by enhancing its antioxidant system, including higher activity of APX and SOD, and higher accumulation of quaternary ammonium compounds (QACs), when compared to CC. However, the sudden increment of ROS production observed in *Citrus* leaves and roots under B deficiency and B excess conditions might be of great biological benefit, as the signaling advantage of ROS is their close connection to cellular homeostasis and metabolism. Almost any change in intracellular homeostasis may lead to a change in the steady-state level of ROS in a specific compartments [57].

Highly mobility of B from mature leaves to tender leaves and florets, and the existence of B compounds in extra floral nectar and the phloem sap, validated that sugar alcohols, such as sorbitol and mannitol, could facilitate the pholem mobility in sorbitol-rich species within *Prunus*, *Pyrus*, and *Malus* [31,33,37,42]. Gene-modified enhancement of sorbitol biosynthesis accompanied by an increased B concentration indicated that sugar alcohol can result in increased B uptake in plants [37,42]. So far, the metabolism of sugar alcohols in *citrus* rootstocks was still unknown.

Several studies showed that *Citrus* rootstocks differed in physiological responses under varied B conditions [22,36,65,66,67]. ‘Newhall’ orange (*C. sinensis*) scion grafted on CC showed higher tolerance of long-term B deficiency than that grafted on trifoliate orange, which was demonstrated by the fact that low-B decreased leaf CO_2_ assimilation, stomatal conductance and transpiration rate but increased intercellular CO_2_ concentration in ‘Newhall’ orange leaves. Furthermore, there existed a fine-tuning distribution of B in citrus roots and shoots, as B in scion and rootstock showed a restricted translocation of B to shoots under conditions of limited B availability [66]. Subsequent study found that different regulation of metabolic pathways about chlorophyll decomposition, glucose synthesis, lignin and carotenoid synthesis might be of great influence on the distinct responses of ‘Newhall’ orange scion grafted on CC and trifoliate orange [22]. Moreover, B was preferentially transferred to upper–younger leaves to support their growth, implying an unveiled mechanism in sensing and balancing the B status between roots and shoots in *Citrus* plants [66].

In *Citrus* plants, not only did total B content affect and determine plant growth and development, the speciation of B in *Citrus* could also influence the physiological responses in different scion-rootstock combinations. For instance, the ratio of semi-bound B to free B (semi-bound B/free B) was higher in citrange-grafted ‘Newhall’ navel orange than in trifoliate orange-grafted plants under the same B supply level. These results demonstrated that citrange-grafted plants had a higher B utilization efficiency than that grafted on trifoliate orange [67]. Moreover, *Citrus* plants could strategically regulate the mineral nutrient concentrations in responding to the abnormal B status. For example, Verna lemon trees grafted on CM rootstock had a higher concentration of nitrogen (N) in the leaves and roots than those grafted on CC, CL, SO. According to previous study that improving N fertilization can alleviate B stress by enhancing the antioxidant system in lentils and barley, the increase in N concentration might have helped to mitigate the adverse effects of a higher concentration of B in the leaves and roots of Verna lemon scions [36,68]. However, investigation of the responses of different rootstock seedlings under B excess revealed that SO rootstock was the most tolerant species to B excess, while CC was the most sensitive ones [65]. The possible mechanism underlying the higher tolerance of SO to B excess might be attributed to the restriction of the uptake and transport of B towards the aerial part, and higher activities of antioxidant enzymes, such as APX and SOD [65]. These results indicated that interaction of scion and rootstock might implicate in transportation and re-distribution of B, and physiological responses of scion under B stress conditions. In conclusion, both low B and B excess led to decomposition of Chls, decreased CO_2_ assimilation, provoked ROS production and ROS scavenging system, and eventually inhibited *Citrus* tree growth and fruit production.

## 3. Molecular Responses to B Stress in *Citrus*

In recent decade, in order to better understanding the adaptive mechanisms of plants to B stresses, many efforts have been made to investigate the molecular response of plants to B nutrition disorder. Evidence shows that the expression levels of genes related to B uptake and translocation, carbohydrate and energy metabolism, cell wall and membranes, N metabolism, stress responses, nucleic acid metabolism, signal transduction, development regulation and cell cycle, were altered by B stresses in *Citrus* plants [17,29,33,34].

By using iTRAQ technique, a total of 164 up-regulated and 225 down-regulated proteins were identified in B-deficient *C. sinensis* roots. These differentially abundant proteins were grouped into the following functional categories: protein metabolism, nucleic acid metabolism, carbohydrate metabolism, cellular transport, cell wall and cytoskeleton metabolism, signal transduction, stress responses, and lipid metabolism [17]. The adaptive responses of *Citrus* roots to B deficiency might include several altered physiological processes, such as decreasing root respiration, improving the total ability to scavenge ROS, and enhancing cell transport. As we mentioned above that B stresses disturbed ROS equilibrium in cellular level in *Citrus*, B deficiency increased the abundance of 31 proteins related to antioxidant system including SOD, POD, MDAR, aldehyde dehydrogenase (ALDH), CBS family protein, hemoglobin1 and HSPs/chaperones in *C. sinensis* roots [17]. B deficiency also induced oxidative stress in *C. grandis* roots as the abundances of many antioxidant proteins such as small ubiquitin-like modifier, peroxiredoxin, peroxidase 2, glutathione peroxidase, methionine sulfoxide reductase, cystatin B, dehydrin family protein, MLP-like protein and antioxidant 1 were increased [4].

In order to gain the comprehensive view of transcriptomic profile in *Citrus* plants under low B and B excess conditions, the cDNA-AFLP technique was used to investigate the differentially expressed genes and their related biological processes of *C. grandis* and/or *C. sinensis* under B stress [30,31,32]. A total of 82 and 83 differentially expressed transcript-derived fragments (TDFs) from leaves and roots were isolated in *C. sinensis* under B deficiency, respectively. These TDFs were mainly involved in amino acid metabolism, energy metabolism, nucleic acid metabolism, cell transport, signal transduction, and stress response. Most of the TDFs associated with signal transduction and stress response were down-regulated in *C. sinensis* roots, but up-regulated in leaves. TDFs related to protein ubiquitination and proteolysis were mostly induced in B-deficient leaves. These results proposed a new insight into the different adaptive mechanisms of *C. sinensis* roots and leaves to B deficiency at the transcriptional level [30]. In another study, cDNA-AFLP analysis showed that the variation of transcriptomic profile was larger in *C. grandis* than in *C. sinensis*, as 132 and 68 differentially expressed TDFs were successfully isolated in B-toxic *C. grandis* and *C. sinensis*, respectively. The differentially expressed TDFs were involved in broad physiological processes from signal transduction and photosynthesis to morphological reconstruction. The authors attributed the higher B-toxicity tolerance of *C. sinensis* to higher expression levels of genes involved in photosynthesis, light utilization and ROS scavenging ability in leaves, which preventing them from photo-oxidative damage [31]. Similar result was also found in *Citrus* roots, where 72 and 110 differentially expressed TDFs were isolated from excess B-treated *C. sinensis* and *C. grandis* roots, respectively. The higher B-toxicity tolerance of *C. sinensis* could be explained by the differentially expressed genes involved in root hair development, antioxidant system, Ca^2+^-mediated signal transduction, and lipid metabolism between *C. sinensis* and *C. grandis* roots under B excess condition [32]. miRNAs have been identified as important post-transcriptional regulators of gene expression in plants. From this point, Lu et al. [29,35] used Illumina sequencing to investigate the effects of B deficiency on the expression patterns of miRNAs in *C. sinensis* leaves and roots. A total of 172 and 134 differentially expressed miRNAs were identified in B-deficient *C. sinensis* leaves and roots, respectively. The response of miRNA profile to B deficiency which contributes to low-B tolerance in *C. sinensis* leaves, might include several aspects: (a) the attenuation of plant growth and development by repressing auxin signaling (miR393, miR160 and miR3946); (b) maintaining leaf phenotype and enhancing the stress tolerance (miR159, miR782, and miR3946, etc.); (c) activation of the stress responses and antioxidant system (miR164, miR6260 and miR5929, etc.); and (d) lowering B export (miR5037) [35]. In contrast, the possible roles of miRNAs in the tolerance of roots to B deficiency could be summarized as: (a) inactivating ROS signaling and scavenging (miR474, miR782 and miR843); (b) increasing lateral root number (miR5023); (c) enhancing cell transport (miR830, miR5266 and miR3465); and (d) improving osmoprotection (miR474) [29]. Furthermore, Sang et al. [27,28] investigated the differentially expressed protein profiles of two *Citrus* species differing in B-toxicity tolerance, and found that B excess increased the abundances of protein species involved in antioxidation, detoxification, proteolysis and cell transport in leaves and roots of *C. sinensis* more than those of *C. grandis*. Although B excess decreased photosynthesis rate in both *C. sinensis* and *C. grandis*, various differentially abundant proteins in leaves conferred a better maintenance of energy homeostasis to *C. sinensis* [27]. In roots, the differentially abundant proteins related to the methyl cycle, the cell wall and cytoskeleton integrity, and the detoxification of the ROS and other toxic compounds such as aldehydes, might be responsible for the higher B-toxicity tolerance of *C. sinensis* [28]. 

For ameliorating the adverse effects of B disorder, some elements or compounds have been applied to plants. For instance, application of fertilizers containing Si, Ca, and Zn, could facilitate photosynthesis of plants grown in excess B [37]. The application of NO, chitosan, MeJA, melatonin and 28-homobrassinolide (HBL) were also reported to improve plant growth and photosynthesis related performance parameters, such as stomatal conductance, net photosynthetic rate, and intercellular CO_2_ concentrationin several crops (*Artemisia annua,* cucumber, and *Vigna radiata*) under B-toxic conditions [37]. Although the underlying mechanisms were not fully elucidated, the application prospect of those elements or compounds in *Citrus* plantation, especially those located in arid and semi-arid area, could be expected in the future. For assistant selection breeding, future efforts could pay attention to some biomarkers or key proteins such as trans-l, 2-diaminocyclohexane-N, N, N,N-tetraacetic acid (CDTA)-soluble pectin, sorbitol-6-phosphate dehydrogenase, and LAC7, which played vital roles in pectin biosynthesis, B transport and lignin biosynthesis in *Citrus* plants under B stress, respectively [18,42,43]. More recently, Huang et al. [45] reported that B-toxicity-induced up-regulation of *CsiLAC4* due to the downregulation of miR397 led to the lignification of the xylem cell walls and the restriction of B flow from xylem vessels to the phloem in *Citrus*.

## 4. Ameliorative Roles of B on Abiotic Stresses in *Citrus*

The prominent role of B in plant cells determined the compact and complex structure of cell wall, and cell wall played as the primary barrier to harmful microorganisms and toxic elements [3,68]. In other crops, for example, in rice and rapeseed, application of B was reported to alleviate cadmium toxicity by decreasing organelle Cd concentration and increasing Cd adsorption on the cell wall, which eventually restricted the entry of Cd ions into the cells and protecting plants from oxidative stress in the roots and leaves of rice [69,70,71]. Interestingly, B could increase root pectin biosynthesis and decrease the degradation of pectin by downregulating the activity of pectinase activity, in addition, the methylation of pectin was also decreased by the higher methylesterase activity to increase the chelation site of Cd onto the cell walls in rapeseed [72]. Apart from increasing Cd being bound to the cell walls and decreasing Cd entering into organelle, elevating B supply could alleviate Cd-induced oxidative stress by increasing the activity of the major antioxidant enzymes, SOD, POD, and CAT [71]. Those results implied that the alleviative effects of B in Cd toxicity not only dependent on regulating constituent and properties of cell wall to facilitate Cd binding, but also include regulating the cellular biological process to flexibly encounter the adverse effects of Cd toxicity and subsequent physiological disorders [71,72,73]. Similarly, the alleviation of Al-induced inhibition in plant growth and development due to elevated B concentration in nutrient solution has been observed in many higher plants, including pea [74], flax [75] and *Citrus* [76,77,78].

Al toxicity and B deficiency are two major factors limiting crop production in tropical and subtropical areas. Our previous study showed that elevating B supply can alleviate the Al-induced inhibition of growth in *C. grandis* [76,77,78]. By using 2-DE based MALDI-TOF/TOF-MS method, sixty-one differentially abundant proteins in *Citrus* roots in response to B-Al interactions were successfully identified [76]. These differentially abundant proteins mainly involved in modification of cell wall, turnover of dysfunctional proteins, stress response, cellular biological regulation and signal transduction. Some proteins including cell division protein 48 (CDC48), calreticulin and phospholipase, might be involved in the downstream signaling of the beneficial effects of B in alleviating Al stress in *Citrus* plants.

## 5. Conclusions

Due to the miniscule requirement of B in crop growth, the essentiality of B in agricultural production is frequently ignored by the production practitioners. The small range of B suitability makes the inappropriate application of B fertilizer a measure at fault leading to B toxicity. Thus, B deficiency and B excess might simultaneously exist at some area. Management of B nutrition became a crucial process, especially in fruit-based industry including *Citrus* production. Visible symptoms of B deficiency and excess include dieback of growing sites, leaf chlorosis and poor performance of photosynthesis system. Physiological analysis showed that B disorders induced the alternation of primary and secondary metabolisms, such as constituent of cell wall, nutrient absorption, antioxidant systems, carbohydrate and energy metabolism. Proteomic and transcriptomic studies showed that B disorder affected the whole process of *Citrus* development from gene transcription to the final morphogenesis. Different scion-rootstock combinations revealed an apparent distinction of B distribution between roots and shoots, and among different *Citrus* species. Future study should focus on the mechanisms underlying the transportation and relocation of B in *Citrus*, especially the metabolism of the companion compounds of B transport, such as sugar alcohols. Furthermore, except for regulating the structure and content of cell wall constituents, the physiological functions of B in other biological processes should also be investigated to better boost the sustainable and high-quality *Citrus* production in the future study.

## Data Availability

Not applicable.
